# Rete-like collateral PICA associated aneurysm surgical resection

**DOI:** 10.1093/jscr/rjag894

**Published:** 2026-10-07

**Authors:** Dar-Yuan Fang, Ming-Chi Kuo

**Affiliations:** Division of Neurosurgery, Department of Surgery, Changhua Christian Hospital, Changhua, Taiwan; Division of Neurosurgery, Department of Surgery, Changhua Christian Hospital, Changhua, Taiwan; Neurosurgery, National Taiwan University Hospital, No. 7, Chung Shan S. Rd. (Zhongshan S. Rd.), Zhongzheng Dist., Taipei 100, Taiwan

**Keywords:** PICA aneurysm, surgical resection, vascular surgery

## Abstract

Rete-like collateral is rare in humans, especially at posterior circulation. This case report explores the direct resection of a ruptured aneurysm associated with the bilateral rete-like collateral artery of posterior inferior cerebellar artery (PICA). The aneurysm was successfully treated by surgical resection. This case highlights the importance of recognizing this unusual vascular anatomy and demonstrates that microsurgical resection of ruptured aneurysm associated with bilateral anomalous posterior inferior cerebellar anastomotic arteries was an effective treatment and careful consideration of the anatomical characteristics concerning the interesting aneurysm and the variant PICA was critical for sate treatment.

## Introduction

Posterior inferior cerebellar artery (PICA) vascular abnormalities such as aneurysms are rare, accounting for <3% of all aneurysm cases [1].

The distinct variations in the fetal development of the arterial network associated with PICA anomalies contribute to diverse pathological features, which significantly influence various vascular disorders within the posterior circulation [2, 3]. One such variation is the presence of rete mirabile or rete-like collateral arteries. Rete mirabile is typically associated with functions such as thermoregulation, pressure regulation, or gas exchange, which is considered a normal anatomical feature. For example, carotid rete mirabile, which is the main feeder for the circle of Willis that regulates pressure, maintains cerebral blood flow, and prevents brain overheating, is a physiological vascular network found in some mammals [4]. It is commonly observed in mammals such as cats, sheep, goats, cows, and pigs [5]. However, in humans, such a structure lacks physiological function and is regarded as either vestigial or pathological in nature, commonly referred to as a rete-like collateral formation [1, 2].

This case report investigates the use of direct resection for a ruptured aneurysm associated with the bilateral rete-like collateral artery of the PICA. In addition, a review of the existing literature on this rare lesion was conducted.

## Case report

A 69-year-old female patient who presented with sudden-onset dizziness, severe headache, and vomiting for >3 hours was brought to our emergency department. Her medical history included spontaneous subarachnoid haemorrhage (SAH), intraventricular hemorrhage, hypertension, and type 2 diabetes mellitus. Upon physical examination, she appeared clear, with a Glasgow Coma Scale score of E4V5M6 and equal pupil sizes of 3.0 mm bilaterally. There was no focal neurological deficit, and the patient was otherwise normal.

The patient underwent a noncontrast-enhanced computed tomography (CT) scan of the brain. Results revealed intraventricular haemorrhage (IVH) in both the lateral ventricles and SAH in the basal cistern and the tentorium of the cerebellum (Fig. 1). No hydrocephalus or midline shift was observed. Subsequently, a three-dimensional digital subtraction angiography was performed, which showed a 5.0 × 3.7-mm saccular aneurysm located at the left PICA (Fig. 2). These findings indicated a ruptured aneurysm located on the rete-like collateral artery connected to the bilateral PICA.

**Figure 1:**
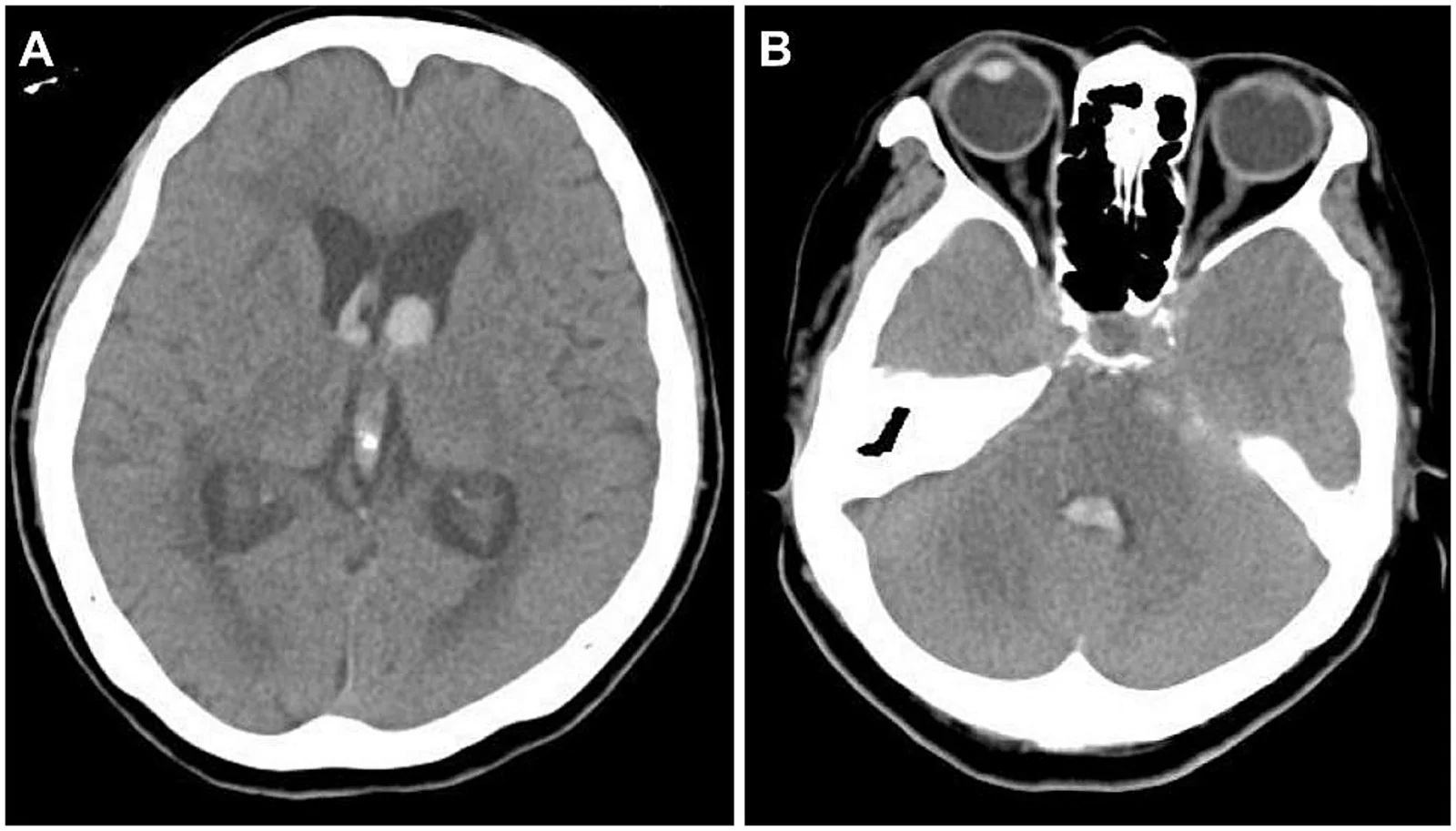
Noncontrast-enhanced CT of the brain demonstrating intraventricular hemorrhage in both lateral ventricles (A) and subarachnoid hemorrhage involving the basal cistern and along the cerebellar tentorium (B).

**Figure 2:**
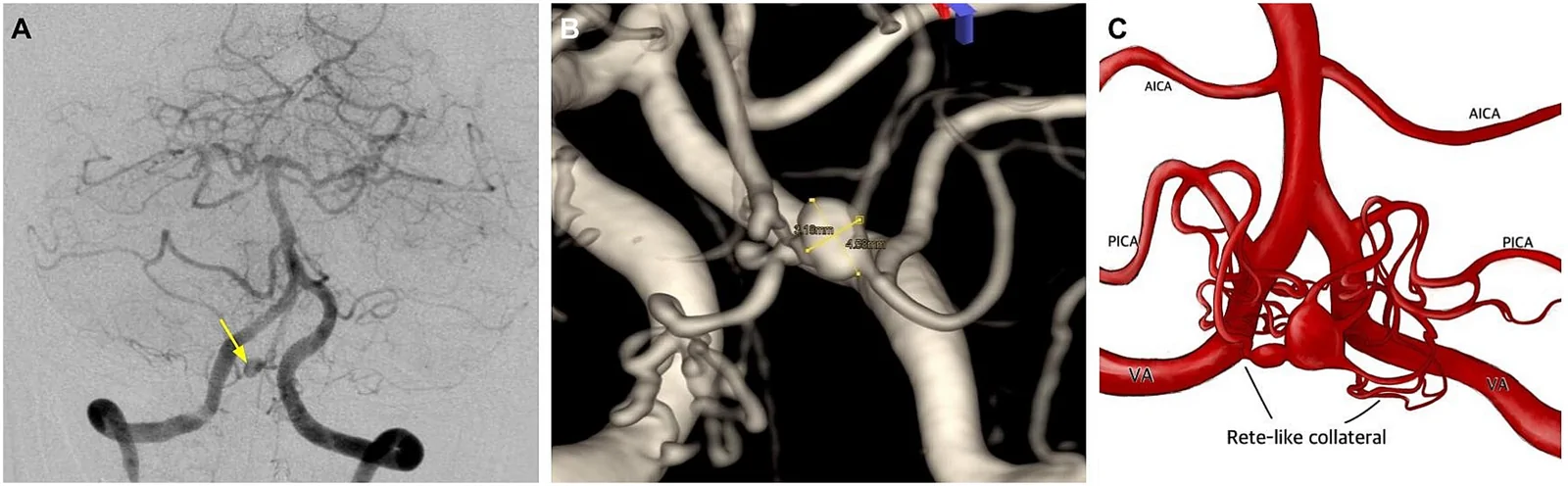
Digital subtraction angiography and three-dimensional imaging showing a 5.0 × 3.7-mm saccular aneurysm at the left posterior inferior cerebellar artery. The aneurysm is identified on digital subtraction angiography (A) and volume-rendered technique imaging (B). Diagram of the aneurysm (C).

Transcatheter arterial embolization (TAE) and open surgical treatment were considered. However, owing to limitations in endovascular devices and distal catheter accessibility, TAE is generally limited to aneurysms located at or proximal to the P2 segment. Therefore, the suboccipital approach was selected to surgically clip the aneurysm.

During the surgery, an external ventricular drain was initially placed through the Frazier’s point to facilitate cerebrospinal fluid drainage. A suboccipital craniectomy was then performed, which created a bone flap with a size of ~6.5 × 4 cm that can expose both cerebellar hemispheres. The arachnoid membrane of the cisterna magna was cautiously opened, and the PICA was identified and meticulously dissected under microscopic visualization (Fig. 3A and B). The intraoperative indocyanine green test was conducted to validate the patency of blood flow from the bilateral PICA to the aneurysm. An aneurysm clip with an appropriate size was selected and applied to secure the aneurysm (Fig. 3C). The aneurysm was successfully resected. The bone flap was then replaced, and the surgical wound was closed in layers.

**Figure 3:**
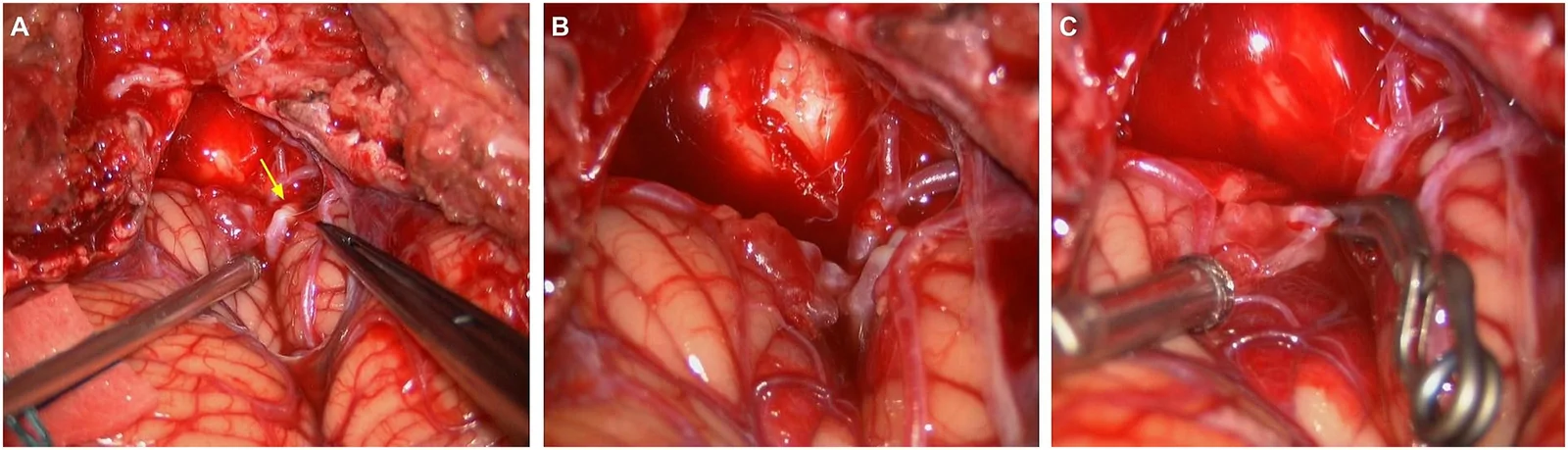
Intraoperative microscopic views demonstrating identification of the posterior inferior cerebellar artery and the aneurysm (A, B). An aneurysm clip was applied to secure the aneurysm (C).

Postoperatively, the patient’s headache and dizziness gradually resolved, without neurological deficits or other complications observed during the recovery period. Postoperative three-dimensional digital subtraction angiography revealed the disappearance of the aneurysm (Fig. 4). During the 1-year follow-up, the patient did not exhibit neurological deficits, with a modified Rankin Scale score of 0.

**Figure 4:**
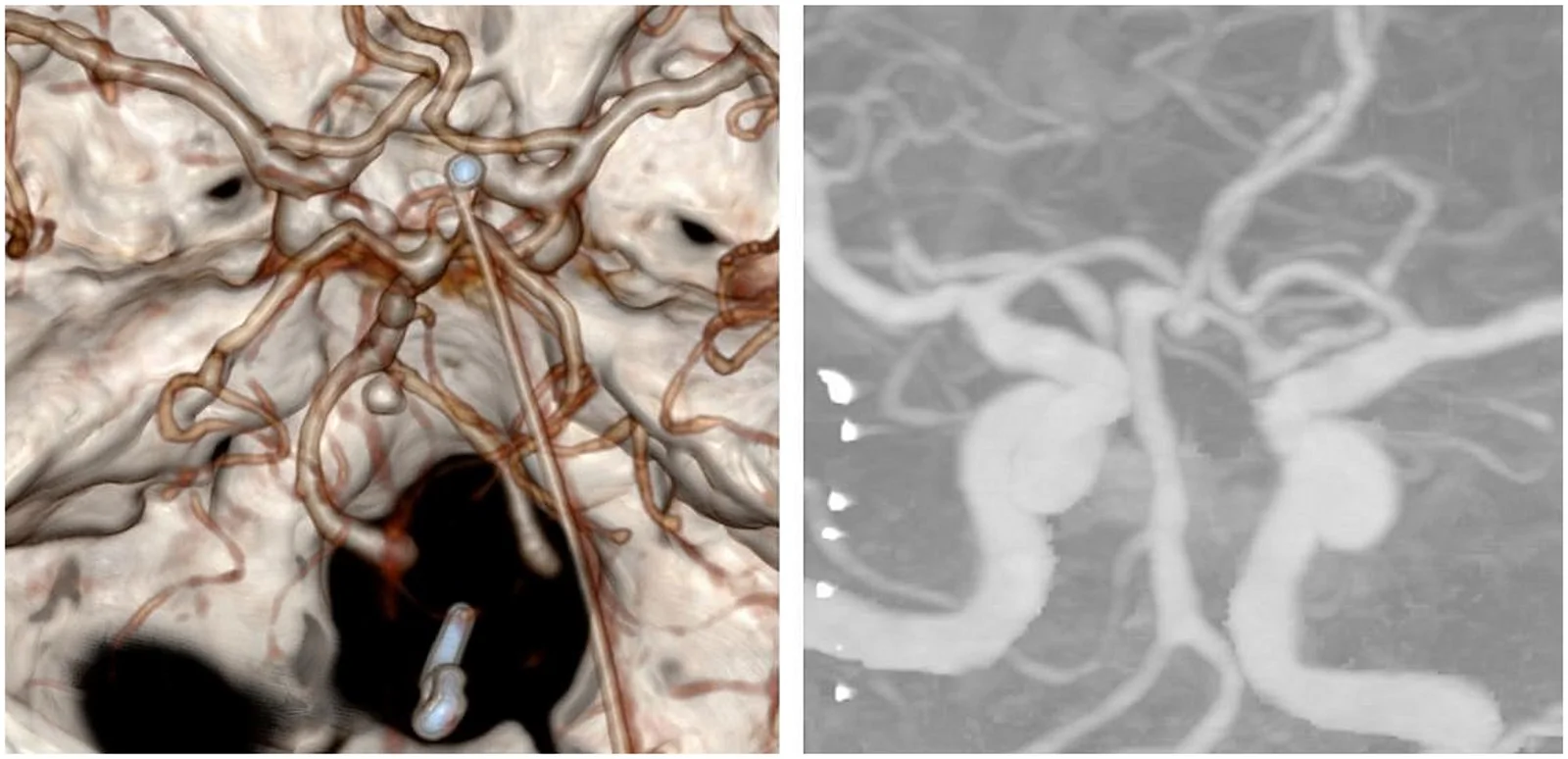
Postoperative three-dimensional digital subtraction angiography demonstrating complete obliteration of the aneurysm without a residual aneurysm sac.

## Discussion

Rete-like collateral may involve both the anterior and posterior circulations, which is associated with conditions such as aneurysm, arteriovenous malformation, carotid cavernous fistula, and vein of Galen aneurysmal malformation, pseudoxanthoma elasticum, and Dieulafoy’s lesion [3, 4]. Rete-like collateral arteries in the posterior circulation can significantly alter collateral blood flow and PICA hemodynamics, potentially predisposing the artery to aneurysm formation [6].

In recent study, EVT is applied in 55.4% of cases of aneurysmal rupture, has become the preferred treatment strategy for PICA aneurysms [7]. However, in patients with PICA–PICA anastomoses, bilateral PICAs initially form anastomoses and collateralization during fetal development. The anomalous anastomotic artery between the bilateral PICAs experienced hemodynamic stress, making it vulnerable to aneurysm formation and rupture, similar to anterior communicating artery aneurysms. Based on the vascular configuration, resection, bypass, or endovascular treatment may be considered to decrease ischemic risks. Therefore, direct surgical resection of the aneurysm was the preferred treatment [2].

In our case, the aneurysm on the bilateral posterior inferior cerebellar anastomotic artery was identified as a low-flow aneurysm, which was confirmed using the intraoperative indocyanine green test. The normal intracranial pressure of the patient was attributed to the low-flow nature of the aneurysm. Patients often present with a normal level of consciousness despite experiencing SAH or stroke. Consequently, bypass was not required during the direct surgical resection.

In conclusion, direct resection of ruptured aneurysms associated with bilateral anomalous posterior inferior cerebellar anastomotic arteries was an effective treatment. Further, an understanding of the unique anatomical characteristics of the aneurysm and the variant PICA should be cautiously considered to ensure a safe and successful management.
